# Supplementary figures and images for: Non-canonical genomic driver mutations of urethane carcinogenesis
Source: PLoS One. 2022 Apr 28;17(4):e0267147. doi: 10.1371/journal.pone.0267147 (PMC9049545; doi:10.1371/journal.pone.0267147)

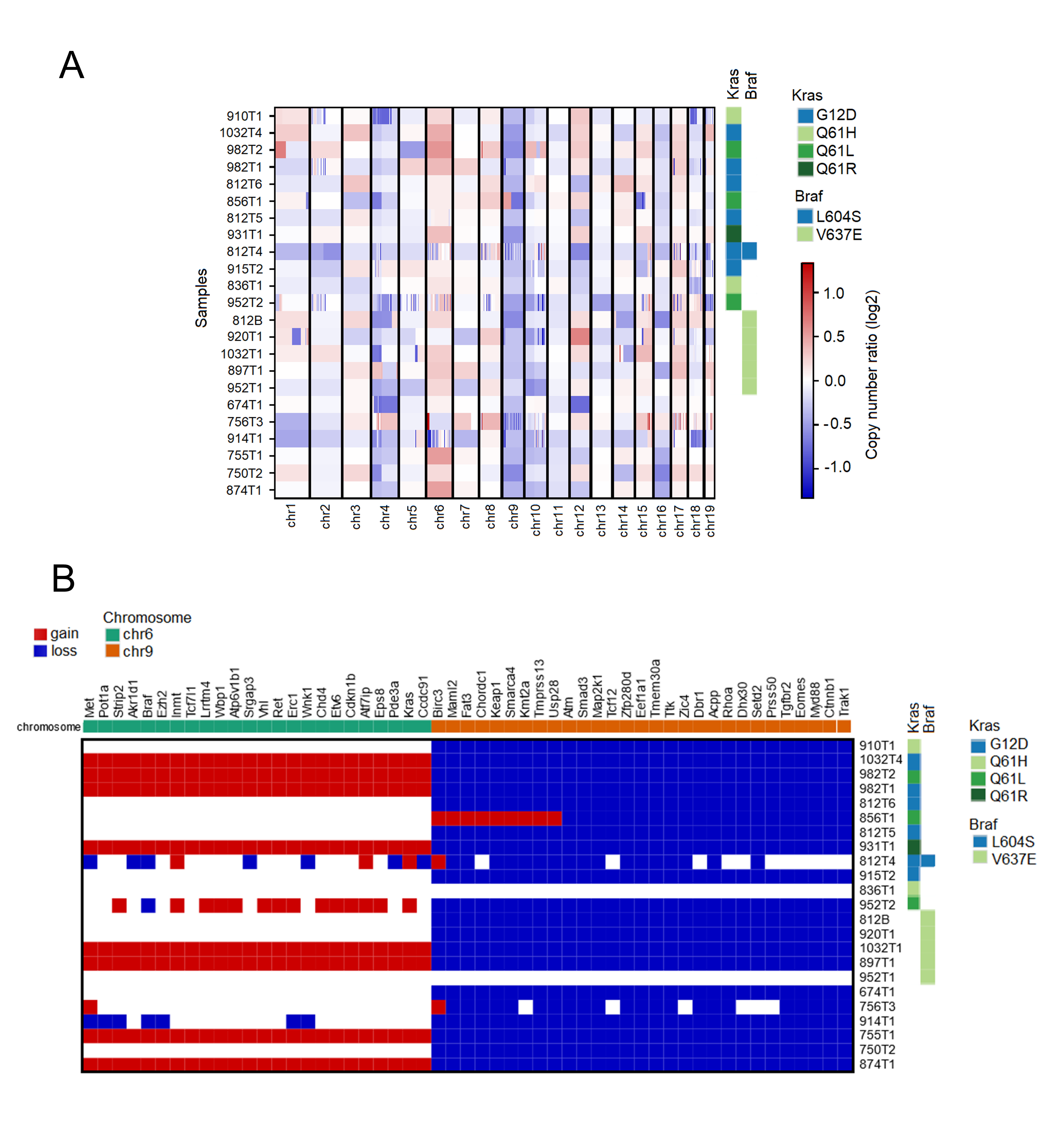

Supplement: S1 Fig — (A) Heatmap of log2-transformed copy number ratios showing areas of genomic gain or loss within each tumor. Columns correspond to genomic bins and rows correspond to individual tumors. Tumors are annotated by the type of Kras or Braf mutations present in the tumor. (B) Cancer-associated genes with copy number gain (log2 ratio > 0.25) or loss (log2 ratio < -0.25) in chromosome 6 and 9 in each tumor. Included genes are from the list of 460 cancer genes examined in Fig 2. (TIF) [file pone.0267147.s001.tif]
