# Supplementary material for: Non-canonical genomic driver mutations of urethane carcinogenesis
Source: PLoS One. 2022 Apr 28;17(4):e0267147. doi: 10.1371/journal.pone.0267147 (PMC9049545; doi:10.1371/journal.pone.0267147)
Supplement: Attachment — Submitted filename: Response to reviewers.pdf [file pone.0267147.s007.pdf]

Reviewer #1:

Comment 1: "Please be careful about the use of the term "driver" without functional validation. The functional validation, such as the possibility of creating transgenic mouse models expressing Rab3gap2 Y467C and KrasG12D, is so exciting, but clearly outside the scope of the current paper. Perhaps the term "genomic driver" would be more accurate."

Response: *As requested*, thank you for this suggestion, we have now replaced the term "driver" with "genomic driver" throughout text.

Comment 2: "The authors could perform copy number analysis (CNVkit) using the whole exome sequencing data generated for this manuscript. This analysis could provide possible genomic drivers for the tumors that don't have an obvious SNV genomic driver."

Response: *As requested*, we performed copy number analysis using CNVkit and identified copy number changes potentially conducive to tumor progression in tumors without obvious SNV genomic drivers (*see* Fig S1 and the Discussion). Thank you for this suggestion!

Comment 3: "Have the authors considered performing subclonal analysis and/or attempting to predict the timing of the genetic events contributing to carcinogenesis in these tumors? This analysis could add depth to the description of urethane carcinogenesis. Please see PLoS Genet 2015 Mar 13;11(3):e1005075. doi: 10.1371/journal.pgen.1005075. eCollection 2015 Mar. for an example of this analysis. WES of the germline in these mice would be required to conduct the analysis

Response: *We deeply apologize* for not being able to perform the clonality and evolutionary history analyses, as we do not have matched germline WES data for these tumors. WE very much now regret not retaining matching normal tissue for this analysis.
